# Fusing a Carbohydrate-Binding Module into the *Aspergillus usamii* β-Mannanase to Improve Its Thermostability and Cellulose-Binding Capacity by *In Silico* Design

**DOI:** 10.1371/journal.pone.0064766

**Published:** 2013-05-31

**Authors:** Cun-Duo Tang, Jian-Fang Li, Xi-Huan Wei, Rou Min, Shu-Juan Gao, Jun-Qing Wang, Xin Yin, Min-Chen Wu

**Affiliations:** 1 School of Biotechnology and Key Laboratory of Industrial Biotechnology, Ministry of Education, Jiangnan University, Wuxi, Jiangsu, China; 2 School of Food Science and Technology, Jiangnan University, Wuxi, Jiangsu, China; 3 School of Pharmaceutical Science, Jiangnan University, Wuxi, Jiangsu, China; 4 Wuxi Medical School, Jiangnan University, Wuxi, Jiangsu, China; Universidad de Granada, Spain

## Abstract

The AuMan5A, an acidophilic glycoside hydrolase (GH) family 5 β-mannanase derived from *Aspergillus usamii* YL-01-78, consists of an only catalytic domain (CD). To perfect enzymatic properties of the AuMan5A, a family 1 carbohydrate-binding module (CBM) of the *Trichoderma reesei* cellobiohydrolase I (TrCBH I), having the lowest binding free energy with cellobiose, was selected by *in silico* design, and fused into its C-terminus forming a fusion β-mannanase, designated as AuMan5A-CBM. Then, its encoding gene, *Auman5A-cbm*, was constructed as it was designed theoretically, and expressed in *Pichia pastoris* GS115. SDS-PAGE analysis displayed that both recombinant AuMan5A-CBM (reAuMan5A-CBM) and AuMan5A (reAuMan5A) were secreted into the cultured media with apparent molecular masses of 57.3 and 49.8 kDa, respectively. The temperature optimum of the reAuMan5A-CBM was 75°C, being 5°C higher than that of the reAuMan5A. They were stable at temperatures of 68 and 60°C, respectively. Compared with reAuMan5A, the reAuMan5A-CBM showed an obvious decrease in *K*_m_ and a slight alteration in *V*_max_. In addition, the fusion of a CBM of the TrCBH I into the AuMan5A contributed to its cellulose-binding capacity.

## Introduction

β-Mannanases (EC 3.2.1.78), abbreviated from β-1,4-D-mannan mannohydrolases, can hydrolyze the internal β-1,4-D-mannosidic linkages of mannans. They could be applied in industrial processes, such as bleaching pulps, depolymerizing anti-nutritional factors in feedstuffs, the production of mannooligosaccharides, and extracting oils from leguminous seeds [1]. To date, many researches have been performed on exploiting novel β-mannanases with good properties, improving their catalytic activities by mutating enzyme-producing strains and optimizing fermentation conditions, as well as producing β-mannanases on an industrial scale [2], [3]. However, the applicability of β-mannanases, exemplified by preparing feedstuffs and bleaching pulps, was limited by their low stability at the high temperature and/or extreme pH. To meet the increasing needs for β-mannanases, more interests are being focused on modifying their molecular structures by means of genetic engineering [4].

Based on the sequence alignment, 25,580 CBMs in the CAZY database (July, 2012) were classified into 64 families. The recognized function of CBMs was to bring enzymes into close vicinity to their substrates by binding carbohydrates [5]. Some studies also demonstrated that the fusion of CBMs into enzymes improved the catalytic activity and/or thermostability. For instances, delignification efficiency of the *Pycnoporus cinnabarinus* laccase for softwood kraft pulp bleaching was improved by fusing a family 1 CBM of the *A. niger* cellobiohydrolase B into the laccase [6]. After two CBMs of the *Thermobifida fusca* and *Cellulomonas fimi* cellulases were fused into the *T. fusca* cutinase, respectively, both fusion cutinases exhibited a dramatic increase of up to 3-fold in the amount of fatty acids released from cotton fiber [7].

Almost all β-mannanases reported have been classified into GH families 5, 26 and 113 (http://www.cazy.org/fam/acc_GH.html) [8]. The family 5 β-mannanases either contain an only CD [9] or, besides a CD, carry an additional CBM located at the C-terminus such as *T. reesei* β-mannanase [10] or at the N-terminus such as *Phanerochaete chrysosporium* β-mannanase [11]. In our previous work, an AuMan5A-encoding gene (*Auman5A*) was cloned and analyzed. The multiple sequence alignment among family 5 β-mannanases displayed that no CBM was found in the AuMan5A [12]. To perfect the AuMan5A’s properties, our present work designed an AuMan5A-CBM by fusing a CBM of the TrCBH I into the AuMan5A. Then, the *Auman5A-cbm* was constructed as it was designed theoretically, and expressed in *P. pastoris* GS115. Moreover, enzymatic properties of the reAuMan5A-CBM and reAuMan5A were analyzed and compared.

## Materials and Methods

### Strains, Vectors and Media

*A. usamii* YL-01-78 and *T. reesei* LW-22, isolated from the soil in China, were used as donors of the *Auman5A* and CBH I-encoding gene (*Trcbh*), respectively. *E. coli* JM109 and pUCm-T (Sangon, Shanghai, China) were used for gene cloning and DNA sequencing. Two recombinant T-vectors, pUCm-T-*Auman5A* and pUCm-T-*Trcbh*, were constructed according to the sequences of the *Auman5A* (GenBank accession: HQ839639) and *Trcbh* (GL985084). *E. coli* DH5α and pPIC9K (Invitrogen, San Diego, CA) were used for the construction of recombinant expression vectors. *E. coli* JM109 and DH5α were cultured in a LB medium. *P. pastoris* GS115 was cultured in a yeast extract peptone dextrose (YPD) medium, and its transformant cultured and induced in following media that were prepared as described in the manual of Multi-Copy *Pichia* Expression Kit (Invitrogen): minimal dextrose (MD), geneticin G418-containing YPD, buffered glycerol-complex (BMGY) and buffered methanol-complex (BMMY).

### Bioinformatics Analysis of the β-mannanase and CBM Sequences

The multiple protein sequence alignment of the CBMs was performed using the ClustalW2 program (http://www.ebi.ac.uk/Tools/msa/clustalw2/). The putative N-glycosylation site of the β-mannanase was located using the NetNGlyc program (http://www.cbs.dtu.dk/services/NetNGlyc/). The Protparam program (http://au.expasy.org/tools/protparam.html) was used for predicting the β-mannanase physicochemical properties. The phylogenetic tree of the CBMs was constructed using both the ClustalW2 program and MEGA 4.0 software. The three-dimensional (3-D) structures of the CBMs were predicted by homology modeling using the MODELLER 9.9 program (http://salilab.org/modeller/).

### *In silico* Design of the Fusion β-mannanase

The candidate CBMs were chosen from the phylogenetic tree, and their 3-D structures were modeled based on a CBM crystal one of the TrCBH I (PDB code: 1CBH). While the 3-D structural information of cellobiose, used as the ligand, was handled using the GlycoBioChem PRODRG (http://davapc1.bioch.dundee.ac.uk/prodrg/submit.html). Then, the interaction between the candidate CBM and cellobiose was predicted by molecular docking (MD) simulation. The binding free energy of the CBM with cellobiose, contrary to their affinity [13], was calculated using the AutoDock 4.2 program (http://autodock.scripps.edu), which combines a rapid binding free energy evaluation through precalculated grids of affinity potentials with a variety of search algorithms to find the most suitable binding position for a ligand on a given macromolecule [14]. Finally, a CBM having the lowest binding free energy was selected, and fused into the AuMan5A forming an AuMan5A-CBM.

### Construction of the Fusion β-mannanase Gene

The *Auman5A-cbm* was constructed by fusing the 3′-end region (*lcbm*) of the *Trcbh*, which encodes both a Ser/Thr/Pro-rich linker and a CBM, into 3′-end of the *Auman5A* by overlapping PCR. The *Auman5A* was amplified from the pUCm-T-*Auman5A* with primers F1 (5′-GAATTCTCCTTCGCCAGCACCTC-3′ with an *Eco*R I site, underlined) and R1 (5′-GAGGGTTGCCGGCACTATCAATAGCAGC-3′). The *lcbm* was amplified from the pUCm-T-*Trcbh* with primers F2 (5′-TGATAGTGCCGGCAACCCTCCCGGCG-3′) and R2 (5′-GCGGCCGCTTACAGGCACTGAGAGTAG-3′, with a *Not* I site, underlined). Using the *Auman5A* and *lcbm* as primers and templates, the first-round overlapping PCR for the *Auman5A-cbm* was performed as follows: a denaturation at 94°C for 4 min; 10 cycles of at 94°C for 30 s, 52°C for 30 s, 72°C for 75 s; an elongation at 72°C for 10 min. Then, F1 and R2 were added to the above PCR system to run the second-round PCR under the same conditions, except 30 cycles and an annealing temperature of 56°C. The amplified target band was cloned into pUCm-T, and transformed into *E. coli* JM109. The recombinant T-vector, pUCm-T-*Auman5A-cbm*, was confirmed by DNA sequencing.

### Transformation of the Recombinant Expression Vectors

The *Auman5A-cbm* and *Auman5A* were excised from the pUCm-T-*Auman5A-cbm* and pUCm-T-*Auman5A* by digestion with *Eco*R I and *Not* I, and inserted into pPIC9K, followed by transforming them into *E. coli* DH5α, respectively. Recombinant expression vectors, pPIC9K-*Auman5A-cbm* and pPIC9K-*Auman5A*, were confirmed by sequencing. The resulting recombinant vectors were separately linearized with *Sac* I, and transformed into *P. pastoris* GS115 using a Gene Pulser Apparatus (Bio-Rad, Hercules, CA).

### Screening and Expression of the *P. pastoris* Transformants

All *P. pastoris* transformants were primarily screened based on their ability to grow on a MD plate, then inoculated successively on G418-containing YPD plates at increasing concentrations of 1.0, 2.0 and 4.0 mg/mL to screen multiple copies of the *Auman5A-cbm* and *Auman5A*, respectively. *P. pastoris* GS115 transformed with pPIC9K vector without any insert was used as the negative control. Expression of the gene *Auman5A-cbm* or *Auman5A* in *P. pastoris* GS115 was performed according to the instruction of Multi-Copy Pichia Expression Kit (Invitrogen) with some modifications [15].

### Enzyme Activity and Protein Assays

β-Mannanase activity was determined by measuring the amount of reducing sugars from locust bean gum (Sigma, St. Louis, MA), using the 3,5-dinitrosalicylic acid (DNS) method as reported previously [15]. One unit (U) of β-mannanase activity was defined as the amount of enzyme liberating 1.0 *µ*mol of reducing sugar equivalent per min under the standard assay conditions (at pH 3.6 and 60°C for 10 min). The protein concentration was measured with the BCA-200 Protein Assay Kit (Pierce, Rockford, IL). Sodium dodecyl sulfate-polyacrylamide gel electrophoresis (SDS-PAGE) was carried out on a 12.5% gel using the reported method [16], and the isolated proteins were visualized by staining with Coomassie Brilliant Blue R-250 (Sigma).

### Purification of the Recombinant β-mannanases

After 96-h induction, the cultured medium of the transformant was centrifuged to remove cells. A total of 60 mL of supernatant was brought to 75% saturation by adding solid ammonium sulfate. The resulting precipitate was collected, dissolved in 6.0 mL of 20 mM Na_2_HPO_4_-NaH_2_PO_4_ buffer (pH 7.0), and dialyzed against the same buffer overnight. The dialyzed solution was concentrated to 1.5 mL by ultrafiltration using a 30-kDa cutoff membrane (Millipore, Billerica, MA), and then loaded onto a Sephadex G-75 column (Amersham Pharmacia Biotech, Sweden; 1.6 × 80 cm), followed by elution with the same buffer at a flow rate of 0.3 mL/min. Aliquots of 1.5 mL eluent only containing the reAuMan5A-CBM or reAuMan5A were pooled and concentrated for further studies.

### Temperature Optimum and Stability Assays

The temperature optima of the reAuMan5A-CBM and reAuMan5A were determined under the standard assay conditions, except temperatures ranging from 50 to 80°C. To estimate the thermostability, the reAuMan5A-CBM or reAuMan5A was incubated at pH 3.6 and various temperatures (45–75°C) for 1.0 h, then the residual enzyme activity was measured under the standard assay conditions. The thermostability here was defined as a temperature, at or below which the residual reAuMan5A-CBM or reAuMan5A activity retained more than 85% of its original activity.

### Enzymatic Kinetic Parameter Assays

Reaction rate (U/mg) of the reAuMan5A-CBM or reAuMan5A was measured under the standard assay conditions, except locust bean gum concentrations ranging from 1.0 to 10 mg/mL. The reaction rate versus the substrate concentration was plotted to confirm whether the catalyzing mode of the reAuMan5A-CBM or reAuMan5A conforms to the Michaelis-Menten equation. The enzymatic kinetic parameters, *K*_m_ and *V*_max_ values, were graphically determined from the Lineweaver-Burk plotting.

### Cellulose-binding Capacity Assay

The cellulose-binding test was performed according to the method [17] with some modifications. The cultured supernatant of the transformant was dialyzed against the 20 mM Na_2_HPO_4_-NaH_2_PO_4_ buffer (pH 7.0), and loaded onto a glass column (0.8 × 10 cm), where 1.0 g crystalline cellulose Avicel PH-101 (Sigma) was filled, at a flow rate of 0.2 mL/min, followed by washing with the same buffer. Total volume of the effluent was scaled. The binding capacity of the reAuMan5A-CBM or reAuMan5A was estimated by measuring the activity of unbound β-mannanase in the effluent.

## Results

### *In silico* Design of the Fusion β-mannanase

**Figure 1:**
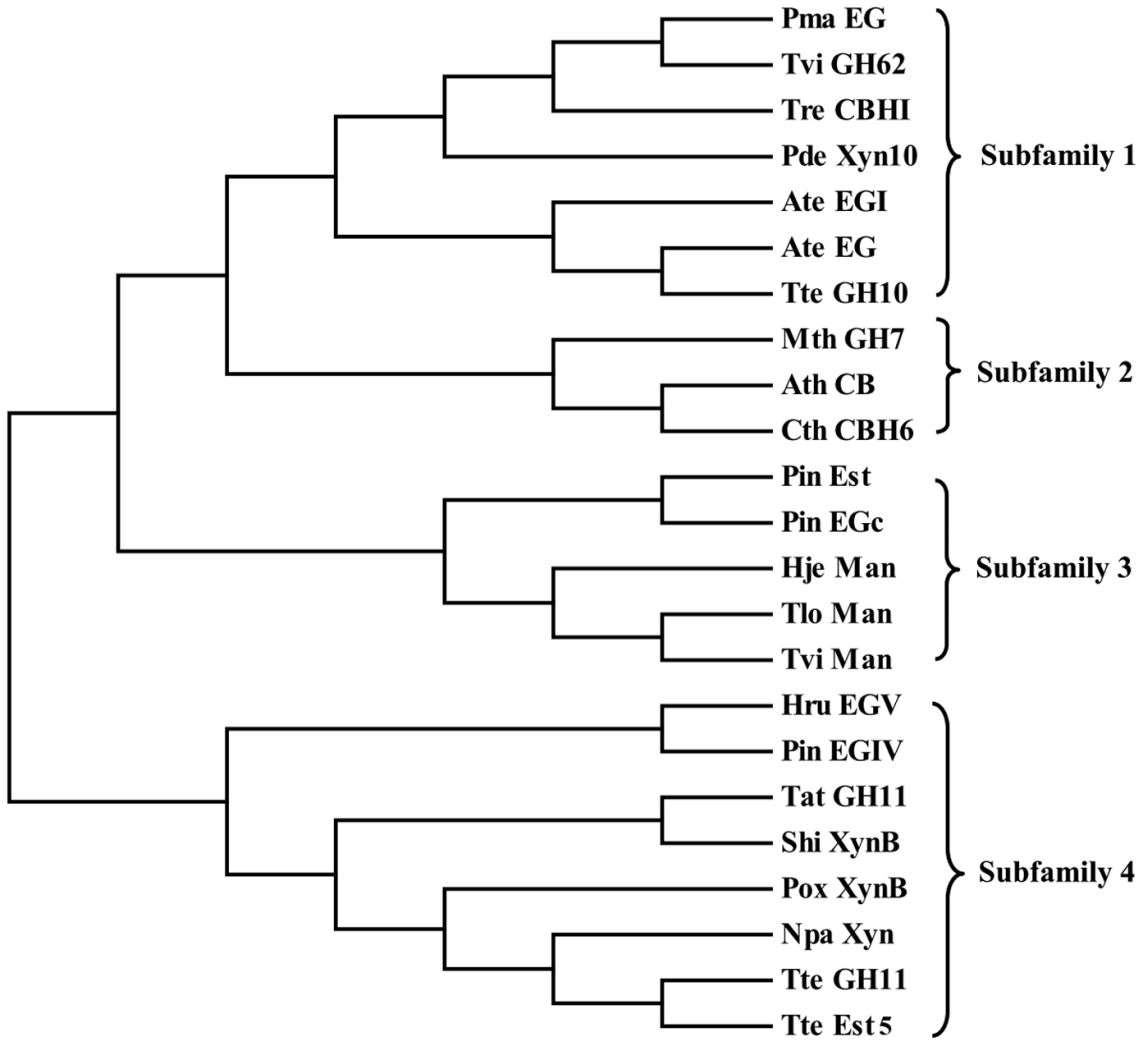
The phylogenetic tree showing the evolutionary relativity and homology among family 1 CBMs. The CBM-containing hydrolases are as follows: Pma EG, *P. marneffei* endoglucanase (XP_002152969); Tvi GH62, *T. virens* GH family 62 enzyme (EHK19840); Tre CBH I, *T. reesei* cellobiohydrolase I (EGR44817); Pde Xyn10, *P. decumbens* family 10 xylanase (ADX86896); Ate EG I, *A. terreus* endoglucanase I (XP_001217291); Ate EG, *A. terreus* endoglucanase (AAW68436); Tte GH10, *T. terrestris* GH family 10 enzyme (XP_003653793); Mth GH7, *M. thermophila* GH family 7 enzyme (XP_003663441); Ath CB, *A. thermophilum* cellobiosidase (CAM98445); Cth CBH6, *C. thermophilum* family 6 cellobiohydrolase (AAY88915); Pin Est, *P. indica* acetylxylan esterase (CCA73570); Pin EGc, *P. indica* endoglucanase c (CCA67649); Hje Man, *H. jecorina* mannanase (AAA34208); Tlo Man, *T. longibrachiatum* mannanase (ADN93457); Tvi Man, *T. viride* mannanase (AFP95336); Hru EG V, *H. rufa* endoglucanase V (AAQ21385); Pin EG IV, *P. indica* endoglucanase IV (CCA70703); Tat GH11, *T. atroviride* GH family 11 enzyme (EHK46770); Shi XynB, *S. hirsutum* xylanase B (EIM91441); Pox XynB, *P. oxalicum* xylanase B (ADV31286); Npa Xyn, *N. patriciarum* xylanase (ABW04217); Tte GH11, *T. terrestris* GH family 11 enzyme (XP_003649436); Tte Est5, *T. terrestris* family 5 carbohydrate esterase (XP_003653797).

**Figure 2:**
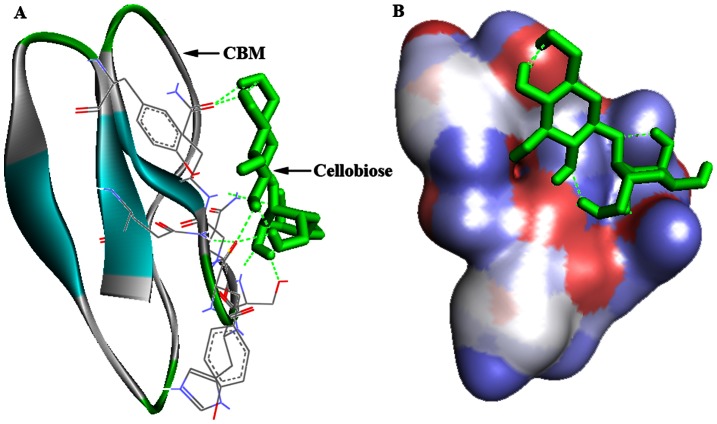
The molecule-docked conformation between 3-D structures of a CBM of the TrCBH I and cellobiose. (A) a cartoon model and (B) a surface model.

From the CAZY database, we randomly selected 23 family 1 CBM sequences. Then, the CBM phylogenetic tree was constructed (Fig. 1). Based on the topology of the tree, those CBMs were further grouped into 4 subfamilies. One to three CBMs from each subfamily were chosen as the candidate CBMs. The interaction between 3-D structures of the candidate CBM and cellobiose was predicted by MD simulation, and then the binding free energy was calculated using the AutoDock 4.2 program (Table 1). As a result, a CBM of the TrCBH I having the lowest binding free energy of −2.27 kcal/mol was selected, and fused into the C-terminus of AuMan5A. The molecule-docked conformation of the CBM with cellobiose was illustrated (Fig. 2).

**Table 1:** The binding free energy of the candidate CBM combining with cellobiose.

CBM-containing hydrolase	GenBank accession no.	Length of CBM (aa)	Aromatic residues in the groove surface	Apolar area/energy of CBM(Å^2^)	Binding free energy (kcal/mol)
Pma EG	XP_002152969	34	Trp^3^, Tyr^29^, Tyr^30^	1775.44	−1.56
Tre CBH I	EGR44817	36	Tyr^5^, Tyr^13^, Tyr^31^, Tyr^32^	1644.23	**−2.27**
Ate EG	AAW68436	33	Trp^10^, Tyr^20^, Trp^28^, Tyr^29^	1780.38	−1.21
Pin Est	CCA73570	33	Tyr^2^, Tyr^10^, Tyr^23^, Trp^27^, Tyr^28^	1753.77	−1.64
Tvi Man	AFP95336	34	Tyr^2^, Tyr^8^, Tyr^23^, Tyr^26^, Trp^27^, Tyr^28^	1776.76	−1.98
Hru EG V	AAQ21385	33	Tyr^2^, Trp^10^, Trp^28^, Tyr^29^	1727.44	−1.65
Pox XynB	ADV31286	20	Trp^15^, Tyr^16^	1275.21	−1.78
Tte GH11	XP_003649436	33	Trp^3^, Trp^11^, Trp^28^, Tyr^29^	1725.45	−1.16

The apolar area/energy of CBM were calculated by the GETAREA servicer (http://curie.utmb.edu/getarea.html).

### Construction of the Fusion β-mannanase Gene

**Figure 3:**
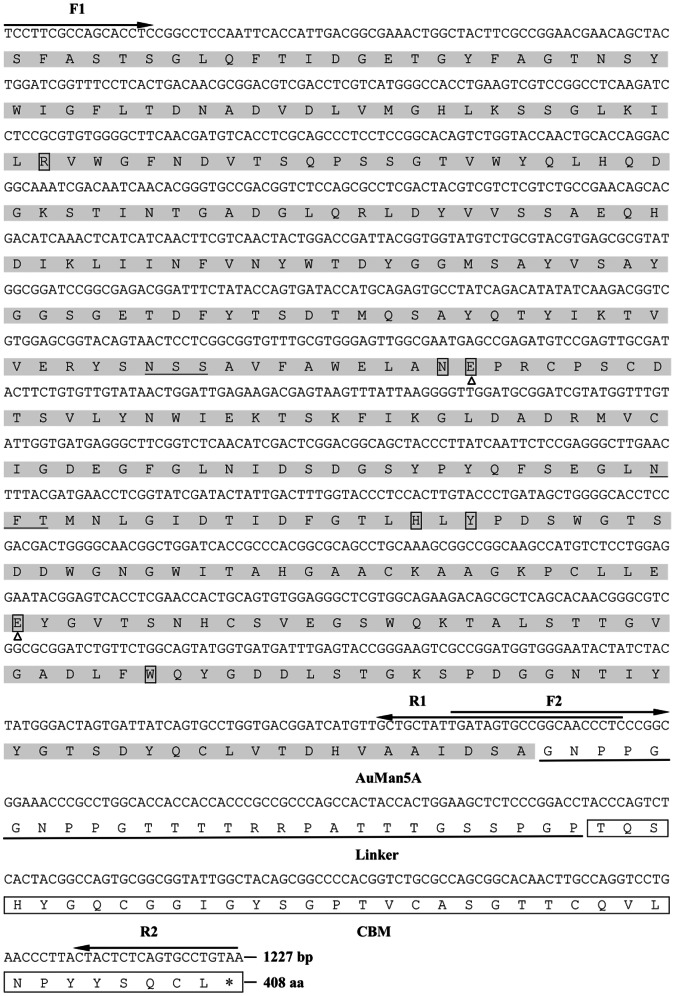
Nucleotide sequence of the *Auman5A-cbm* and its deduced amino acid sequence of the AuMan5A-CBM. The amino acid residues of the AuMan5A are marked in grayed background. A linker of the *T. reesei* CBH I is underlined and its CBM is boxed. Two triangles below the boxed letters indicate the catalytic residues (E168 and E276) and five active site residues (R52, N167, H241, Y243, and W306) are located in grayed boxes. Two putative N-glycosylation sites of the AuMan5A-CBM are underlined in grayed background. The bold arrows above the letters represent the PCR primers.

About 1050- and 210-bp bands of the *Auman5A* and *lcbm* were amplified from the pUCm-T-*Auman5A* and pUCm-T-*Trcbh*, respectively. Then, they were subjected to the overlapping PCR to construct the fusion gene. As a result, an about 1250-bp band of the *Auman5A-cbm* was amplified and inserted into pUCm-T. DNA sequencing result verified that the cloned *Auman5A-cbm* is 1241 bp in length (containing *Eco*R I and *Not* I sites), encoding an AuMan5A-CBM of 408 amino acid residues with a theoretical molecular mass of 43,798 Da and a pI of 4.22. The AuMan5A-CBM consists of a 345-aa CD from the AuMan5A, and both a 27-aa linker and a 36-aa CBM from the TrCBH I (Fig. 3).

### Screening and Expression of the *P. pastoris* Transformants

A total of 30 *P. pastoris* transformants with *Auman5A* respectively resistant to 1.0, 2.0 and 4.0 mg/mL of geneticin G418, numbered as *P. pastoris* GSAuM1-1 to GSAuM1-10, GSAuM2-1 to GSAuM2-10 and GSAuM4-1 to GSAuM4-10, and 30 transformants with *Auman5A-cbm*, numbered as *P. pastoris* GSAuMC1-1 to GSAuMC1-10, GSAuMC2-1 to GSAuMC2-10 and GSAuMC4-1 to GSAuMC4-10, were picked out for flask expression tests. *P. pastoris* GS115 transformed with pPIC9K, numbered as *P. pastoris* GSC, was used as the negative control. After induction by adding 1.0% (v/v) methanol at 24 h intervals for 96 h, the cultured supernatants were used for β-mannanase activity assay. As a result, two transformants with the highest reAuMan5A-CBM and reAuMan5A activities of 40.6 and 44.1 U/mL, numbered as *P. pastoris* GSAuMC4-5 and GSAuM4-8, were selected, respectively. No β-mannanase activity of *P. pastoris* GSC was detected under the same expression conditions.

### Purification of the Recombinant β-mannanases

**Figure 4:**
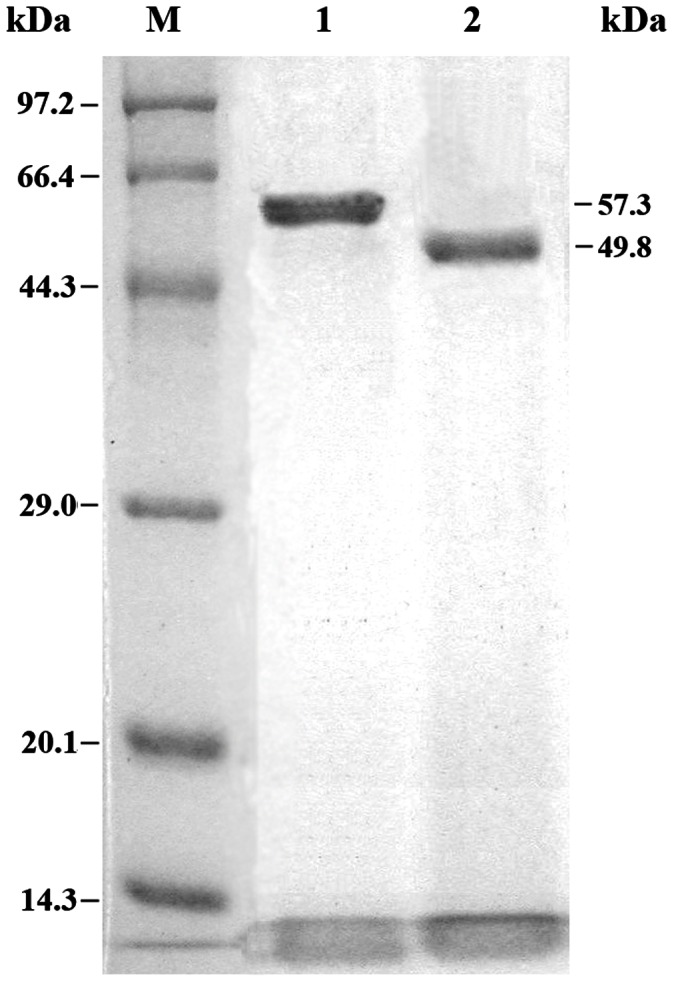
SDS-PAGE analysis of the purified reAuMan5A-CBM and reAuMan5A. Lane M, standard protein marker; lane 1, the AuMan5A-CBM with an apparent molecular mass of 57.3 kDa; lane 2, the reAuMan5A with an apparent molecular mass of 49.8 kDa.

The amount of the reAuMan5A-CBM or reAuMan5A in the cultured supernatant of the GSAuMC4-5 or GSAuM4-8 accounted for more than 85% of that of the total protein. So they were purified to homogeneity only by a simple combination of ammonium sulfate precipitation, ultrafiltration and Sephadex G-75 gel filtration (Fig. 4). Specific activities of the purified reAuMan5A-CBM and reAuMan5A, towards locust bean gum under the standard assay conditions, were 312.3 and 341.3 U/mg, respectively.

SDS-PAGE analysis of the purified reAuMan5A-CBM and reAuMan5A showed two single protein bands with apparent molecular masses of 57.3 and 49.8 kDa, respectively (Fig. 4), which were much larger than their respective theoretical ones (43,798 and 37,614 Da). *P. pastoris* enables some post-translational modifications, including the assembly of disulfide bond, exclusion of the signal peptide, and N-glycosylation, etc. Analysis showed that there are two putative N-glycosylation sites (N156-S157-S158 and N225-F226-T227) in the AuMan5A-CBM or AuMan5A sequence (Fig. 3). To verify whether the difference between apparent and theoretical molecular masses was resulted from N-glycosylation, The N-deglycosylation assay was performed using an endoglycosidase H (New England Biolabs, Ipswich, MA) according to the manufacturer’s instruction. As a result, molecular sizes of the N-deglycosylated reAuMan5A-CBM and reAuMan5A on SDS-PAGE (data not shown) were good agreement with their respective theoretical ones. So the larger apparent molecular masses may be caused by N-glycosylation. Carbohydrate contents of the purified reAuMan5A-CBM and reAuMan5A were determined to be 19.8 and 21.3%, respectively, using the phenol-sulfuric acid method [18].

### Enzymatic Properties of the Recombinant Enzymes

**Figure 5:**
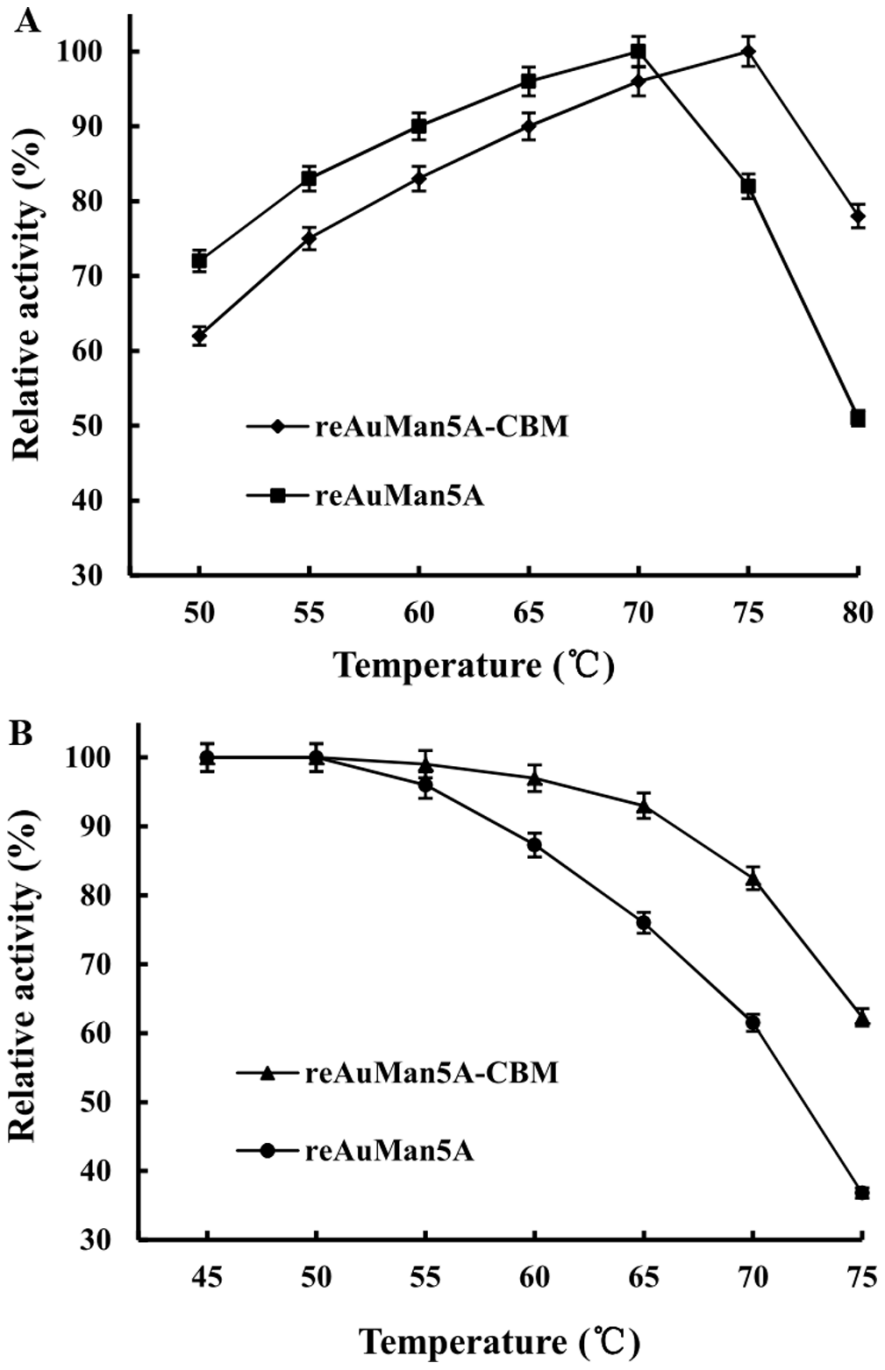
Effects of temperature on (A) activity and (B) stability of the reAuMan5A-CBM and reAuMan5A. Temperature optima of the reAuMan5A-CBM and reAuMan5A were determined, respectively, under the standard assay conditions, except temperatures ranging from 50 to 80°C. Their thermostabilities were evaluated, respectively, by incubating them at pH 3.6 and various temperatures from 45 to 75°C for 1.0 h, and the residual enzyme activities were measured under the standard assay conditions.

The temperature optima of the reAuMan5A-CBM and reAuMan5A were 75 and 70°C (measured at pH 3.6), respectively (Fig. 5A). They were highly stable at temperatures of 68 and 60°C, respectively, but over which their residual activities declined rapidly and only retained 62.3 and 36.8% of the original ones at 75°C, respectively (Fig. 5B).

The *K*_m_ and *V*_max_ values of the reAuMan5A-CBM were determined to be 0.66 mg/mL and 389.1 U/mg, respectively, and those of the reAuMan5A were 1.36 mg/mL and 415.8 U/mg, respectively. The reAuMan5A-CBM displayed an obvious decrease in *K*_m_ but a slight alteration in *V*_max_, as compared with the reAuMan5A.

### Cellulose-binding Capacity

A total of 20 mL of dialyzed cultured supernatant, in which the reAuMan5A-CBM or reAuMan5A activity was adjusted to 20 U/mL, was loaded onto the cellulose column. The test results showed that cellulose-binding capacity of the reAuMan5A-CBM with Avicel PH-101 was up to 92.3%, whereas that of the reAuMan5A was not detected.

## Discussion

Mannans, the major hemicelluloses in plant cell walls and the specific substrates for β-mannanases, are always cross-linked with various kinds of carbohydrates, such as celluloses, xylans and arabinans [19]. CBMs often existed in some hydrolases decomposing plant cell walls, such as β-mannanases, xylanases and arabino furanosidases. Being natively in enzymes or by fusing CBMs into the N- or C-termini of other enzymes, the CBMs could obviously enhance the catalytic efficiency of enzymes by increasing their local concentrations around the polysaccharide substrates, and/or improve the thermostability [17]. Because the AuMan5A consists of an only CD, it is reasonable to perfect its enzymatic properties, such as thermostability and cellulose-binding capacity, by fusing a CBM into the AuMan5A. However, there are a total of 25,580 CBMs classified into 64 families in the CAZY database, so the choice of a suitable CBM from so numerous CBMs is time-consuming and laborious. It was reported that the family 1 CBMs are mainly cellulose-binding modules, while celluloses are often covalently and non-covalently attached to mannans [5]. Therefore, our present work was focused on the family 1 CBMs.

The MD simulation can rapidly predict the most suitable binding position for a ligand on a given macromolecule and calculate the binding free energy, which was used as a powerful tool in indirectly reflecting the affinity between ligand and receptor, such as the design of human phospholipase A_2_ or HIV-1 protease inhibitors [20], [21]. In this work, we selected 23 family 1 CBMs to construct the phylogenetic tree, from which 8 candidate CBMs were chosen. And their 3-D structures were modeled based on a CBM crystal one of the TrCBH I. Next, the interaction between 3-D structures of the candidate CBM and cellobiose was predicted, and the binding free energy was calculated. And finally, a CBM having the lowest binding free energy was selected, and then the *Auman5A-cbm* was constructed and expressed in *P. pastoris* GS115. As can be seen from Fig. 2, several aromatic amino acids are exposed to the groove surface of a CBM of the TrCBH I. In recent years, the 3-D structures of representative members from 22 CBM families have been resolved. Data from these 3-D structures speculated that these aromatic amino acids may play a key role in binding cellobiose or carbohydrates [22], which needs to be proved by our further studies.

*P. pastoris* transformant that can resist a higher concentration of G418 might have multiple copies of integration of a heterologous gene into *P. pastoris* genome, which could potentially result in a high expression level of a heterologous protein as explained in the manual of Multi-Copy Pichia Expression Kit (Invitrogen). However, the expression level was not directly proportional to the concentration of G418 [9], [23]. Due to those reasons, in this work, a total of 30 transformants with *Auman5A-cbm* resistant to different concentrations of G418 and 30 transformants with *Auman5A* were picked out for flask expression tests. This screening procedure has been applied to conduct the over-expression of other recombinant proteins or enzymes in *P. pastoris* [24], [25].

The *P. pastoris* expression system has many advantages, one of which is that the purity of expressed recombinant proteins or enzymes is high. It was reported that the purity of recombinant *A. sulphureus* β-mannanase expressed in *P. pastoris* X-33 was 97% [9]. In this work, the purity of reAuMan5A-CBM or reAuMan5A was more than 85%. So they were purified to homogeneity only by a simple combination of ammonium sulfate precipitation, ultrafiltration and Sephadex G-75 gel filtration (Fig. 4). The purified reAuMan5A-CBM and reAuMan5A were N-glycosylated proteins, which were confirmed by N-deglycosylation and carbohydrate content assays.

The temperature optimum and stability of the reAuMan5A-CBM were 75 and 68°C, which were 5 and 8°C higher than those of the reAuMan5A, respectively, indicating that the fusion of a CBM of the TrCBH I conferred the elevated tolerance to high temperature on the AuMan5A. The *K*_m_ value (0.66 mg/mL) of the reAuMan5A-CBM was much lower than that (1.36 mg/mL) of the reAuMan5A, implying that the former has a higher affinity towards locust bean gum. The cellulose-binding capacity of the reAuMan5A-CBM with Avicel PH-101 was up to 92.3%, suggesting that it can be gathered around the natural substrate (that is, mannan cross-linked with cellulose, xylan and arabinan) by exclusively binding cellulose [26].

### Conclusions

In this work, the fusion β-mannanase gene, *Auman5A-cbm*, was constructed as it was designed theoretically, and functionally expressed in *P. pastoris* GS115. The fusion of a family 1 CBM of the TrCBH I into the C-terminus of AuMan5A obviously improved its thermostability and cellulose-binding capacity, which are beneficial for industrial applications. The superior properties of the reAuMan5A-CBM make it a good candidate in industrial processes. To our knowledge, this work first established a novel strategy for molecular modification of enzymes by *in silico* design.
